# Comparative Influence of Ocean Conditions on Yellowfin and Atlantic Bluefin Tuna Catch from Longlines in the Gulf of Mexico

**DOI:** 10.1371/journal.pone.0010756

**Published:** 2010-05-28

**Authors:** Steven L. H. Teo, Barbara A. Block

**Affiliations:** 1 Department of Wildlife, Fish, and Conservation Biology, University of California Davis, Davis, California, United States of America; 2 Tuna Research and Conservation Center, Hopkins Marine Station, Stanford University, Pacific Grove, California, United States of America; University of Canterbury, New Zealand

## Abstract

Directed fishing effort for Atlantic bluefin tuna in the Gulf of Mexico (GOM), their primary spawning grounds in the western Atlantic, has been prohibited since the 1980s due to a precipitous decline of the spawning stock biomass. However, pelagic longlines targeted at other species, primarily yellowfin tuna and swordfish, continue to catch Atlantic bluefin tuna in the GOM as bycatch. Spatial and temporal management measures minimizing bluefin tuna bycatch in the GOM will likely become important in rebuilding the western Atlantic bluefin stock. In order to help inform management policy and understand the relative distribution of target and bycatch species in the GOM, we compared the spatiotemporal variability and environmental influences on the catch per unit effort (CPUE) of yellowfin (target) and bluefin tuna (bycatch). Catch and effort data from pelagic longline fisheries observers (1993–2005) and scientific tagging cruises (1998–2002) were coupled with environmental and biological data. Negative binomial models were used to fit the data for both species and Akaike's Information Criterion (corrected for small sample size) was used to determine the best model. Our results indicate that bluefin CPUE had higher spatiotemporal variability as compared to yellowfin CPUE. Bluefin CPUE increased substantially during the breeding months (March-June) and peaked in April and May, while yellowfin CPUE remained relatively high throughout the year. In addition, bluefin CPUE was significantly higher in areas with negative sea surface height anomalies and cooler sea surface temperatures, which are characteristic of mesoscale cyclonic eddies. In contrast, yellowfin CPUE was less sensitive to environmental variability. These differences in seasonal variability and sensitivity to environmental influences suggest that bluefin tuna bycatch in the GOM can be reduced substantially by managing the spatial and temporal distribution of the pelagic longline effort without substantially impacting yellowfin tuna catches.

## Introduction

Managing and mitigating the bycatch of non-target species is arguably one of the most pressing issues facing fisheries management agencies and the commercial fishing industry today [1]. Bycatch is generally considered as the mortality induced on non-target species. Recently, there has been increasing focus on reducing bycatch from pelagic longlines targeting tuna and swordfish [1], [2], [3], [4]. The objective of this research is to examine how to minimize bycatch while maximizing the catch of target species at a specific effort level and minimizing mitigation costs. Two of the main mitigation strategies have been changes in the fishing gear and practices, and spatiotemporal management of the fishing effort. For example, relatively simple changes to longline fishing gear and methods, like adding streamers to longlines and setting longlines deep through tubes, have been shown to reduce bycatch of seabirds [5]. However, when gear changes alone are not able to reduce bycatch of the non-target species substantially, spatiotemporal management of the fishing effort may become the strategy of choice [6].

Spatiotemporal management of fishery effort can reduce the interactions of fisheries and bycatch species [6]. In order to be effective, spatiotemporal management requires an understanding of the distributions of both the target and bycatch species and how environmental conditions affect those distributions. Once we understand their habitat preferences, the probability of catching non-target benthic species may be estimated from the benthic habitat of the area [7]. In contrast, the probability of catching non-target pelagic species is estimated from local ocean conditions, which are substantially more dynamic than benthic conditions [6]. Therefore, in order to employ spatiotemporal management for pelagic fish, we need to understand and compare the influence of ocean conditions on the distribution of target and bycatch species.

In recent years, there has been increasing concern about the bycatch of Atlantic bluefin tuna on their spawning grounds in the Gulf of Mexico (GOM) by pelagic longlines targeting yellowfin tuna and swordfish [8], [9], [10]. Bluefin tuna are among the most valuable fishes in the world, with a single Pacific bluefin tuna being sold for 16.28 million yen in 2010 at the Tsukiji market in Tokyo, and an average wholesale price of 3272 yen kg^−1^ in 2007 [11]. As a consequence, the Atlantic bluefin tuna (as well as the other two species of bluefin tuna - Pacific and Southern) are under severe fishing pressure. There are two main spawning grounds and periods for Atlantic bluefin tuna - the GOM from March to June and the Mediterranean Sea from June to August [12], [13], [14], [15]. The International Commission for the Conservation of Atlantic Tunas (ICCAT, http://www.iccat.es) currently manages the Atlantic bluefin tuna as two distinct stocks, with western Atlantic spawners forming a distinct stock from eastern Atlantic spawners. The western Atlantic stock has suffered a >80% decline in spawning stock biomass since 1970 and a 20-year rebuilding plan was enacted in the early 1980s [13]. However, recent assessments indicate that the western stock has continued to decline [16]. One potential factor contributing to the decline of the western stock is the incidental bycatch of spawning bluefin tuna by pelagic longline fisheries in the GOM [8]. The GOM has been closed to directed fishing for bluefin tuna since 1981 [14] but observer and logbook data from the National Marine Fisheries Service (NMFS) and scientific longlining data indicate that there is substantial bycatch of bluefin tuna in the GOM during the breeding season [8]. The pelagic longlines in the GOM generally target yellowfin tuna (the most desirable species) and to a lesser extent, swordfish but Atlantic bluefin tuna are caught as bycatch in the longline sets [10].

In this study, our aim was to compare the influence of ocean conditions on the catch per unit effort (CPUE) of yellowfin tuna and bluefin tuna by pelagic longlines in the Gulf of Mexico, using catch and effort data from the NMFS pelagic longline observer program and scientific cruises. The numbers of bluefin and yellowfin tuna caught on longline sets were fit to negative binomial models, with local environmental conditions as explanatory variables. Count data models, like Poisson and negative binomial models, are often used to model rare event data (i.e., observations are non-negative integers with numerous zeros) [17], which correspond well to the characteristics of our data. Exploration of the longline data indicated that negative binomial models were suitable for analyzing the data because of overdispersion. We also used a model selection process to identify the most important variables that explained the variability in the catch of bluefin and yellowfin tuna [18]. After fitting the models, we used the models to estimate and compare the probability of catching bluefin and yellowfin tuna given ocean conditions in the GOM.

The GOM has distinctive oceanographic conditions, with important differences between the eastern and western portions of the basin. The eastern GOM is dominated by the Loop Current, which flows through the Yucatan Straits and makes an anti-cyclonic turn before exiting through the Florida Straits. In the western GOM, one of the key oceanographic features are cyclonic and anti-cyclonic mesoscale eddies generated by or pinched off from the Loop Current that travel from the east to west [19].

In prior studies, we identified the probable breeding areas and oceanographic preferences of breeding bluefin tuna in the GOM, using electronic tags deployed on mature bluefin tuna [9], [20]. Atlantic bluefin tuna tended to exhibit breeding behavior in the western GOM and the frontal zone of the Loop Current in the central and eastern GOM [9]. Breeding areas used by the bluefin tuna were significantly associated with bathymetry, SST, eddy kinetic energy, surface chlorophyll concentration, and surface wind speed, with SST being the most important parameter [20]. We also used electronic tags to examine the depth and thermal preferences of yellowfin tuna caught on pelagic longlines in the GOM [21]. Yellowfin tuna in the GOM showed a preference for the mixed layer and thermocline, and exhibited a diel pattern in depth distribution, remaining in surface and mixed layer waters at night and diving to deeper waters during the day.

The results from the current study will improve our understanding of the oceanographic habitat utilized by bluefin and yellowfin tuna in the GOM and how changing environmental conditions affect their spatiotemporal distribution. This will in turn help us inform possible spatiotemporal management strategies to reduce the bycatch of bluefin tuna while maintaining yellowfin tuna catches in the GOM. Our results can be used to estimate the probability of bluefin bycatch relative to yellowfin catch in an area given the environmental conditions. Thus, if local environmental conditions in an area are expected to increase the probablity of catching bluefin tuna, longline effort could be directed away from these areas towards areas with lower probability of catching bluefin tuna while maintaining a high CPUE for yellowfin tuna.

## Materials and Methods

### Fishery and Environmental Data

**Figure 1:**
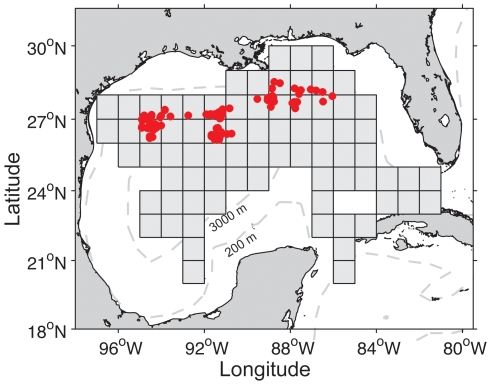
Locations of pelagic longline sets in the Gulf of Mexico. Each grey square indicates where one or more longline sets were recorded by fishery observers on commercial longline vessels (n = 2662 sets) within the 1×1° area (we are unable to show locations of individual sets due to privacy concerns). Each red circle indicates a single longline set during our laboratory's cruises on longline vessels targeting bluefin tuna for tagging (n = 112 sets).

Two sources of fishery data were used for this study. The first consisted of catch and effort data collected by fishery observers on commercial longline vessels in the GOM from 1992 through 2005, as part of the Pelagic Observer Program (POP) managed by NMFS Southeast Fisheries Science Center (n = 2662 sets) (Fig. 1). Although the POP began in 1992 and continues to this day, we only downloaded data from 1992 to 2005 because this was the only data made available by the NMFS. Overall, the average effort for a commercial longline set in the GOM was 755±225 hooks and a soak duration of 9.1±3.3 hours. All information that potentially identified individual fishing vessels or observers were removed from the database prior to analysis in order to minimize any privacy concerns. In addition, we presented observer data in this paper as 1×1° squares to further minimize any privacy concerns (Fig. 1). Further details on the observer program and pelagic longline fisheries in the GOM can be found in the program documentation [10], [22].

The second source consisted of catch and effort data collected by our laboratory during six scientific longline cruises in the GOM from 1998 through 2002, as part of the Tag-A-Giant program effort to tag Atlantic bluefin tuna [8]. Further details on these longline cruises can be found in Block et al. (2005, Supplementary Online Material). These cruises were conducted aboard U.S. registered pelagic longline vessels that routinely fish for yellowfin tuna. Our fishing efforts in the GOM were conducted for the purpose of deploying electronic tags on bluefin tuna and all sets (n = 112) were made in the US exclusive economic zone from 86.06°W to 94.90°W in longitude and 26.67°N to 28.5°N to in latitude (Fig. 1). Circle hooks were baited with squid or sardines and positioned at depths of 100–200 m in 1999 and 40–120 m in 2000–2002. Overall, our longline sets had fewer number of hooks per set (188±95 hooks) and shorter soak durations (2.6±3.6 hours) due to our aim of keeping the bluefin tuna in good condition for tagging. The shorter soak times helped to reduce the stress and mortality rate of the bluefin tuna caught on the longlines [8].

**Figure 2:**
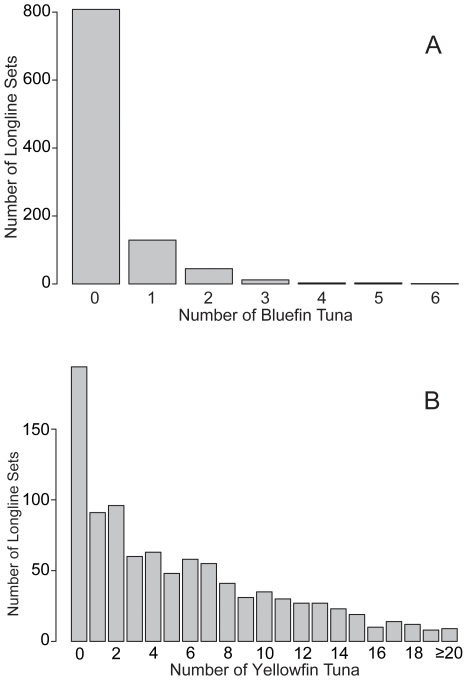
Numbers of bluefin and yellowfin tuna per longline set. Histograms show the number of (A) bluefin and (B) yellowfin tuna caught in each longline set.

Importantly for this study, both data sources included information on the number and species of fish caught (NUMBFT and NUMYFT), year of set (YR), month of set (MTH), set latitude (LAT), set longitude (LON), number of hooks (NUMHKS), and approximate depth of hooks (HKDPTH) for each set, which were used as predictor variables. Definitions and acronyms of the fishery and environmental variables can be found in Table 1. After obtaining the data, we first performed an analysis of the spatial and temporal variability in the catch per unit effort (CPUE, fish per 1000 hooks) of bluefin and yellowfin tuna. The CPUE of both species were first determined by month and in 1×1° squares. Exploratory analyses indicated that most (87.4%) of the observed bluefin bycatch occurred during the bluefin breeding season from March to June. We therefore decided to concentrate our subsequent oceanographic analysis on this important period. After extracting the data for this period, our dataset consisted of 944 longline sets from fishery observers and 112 longline sets from our tagging cruises in the GOM, with a toal of 288 bluefin and 6633 yellowfin tuna. The distribution of the number of bluefin and yellowfin tuna caught in a single set during the breeding period can be seen in Fig. 2. The mean number of bluefin and yellowfin tuna caught in a single set was 0.288±.0493 and 6.63±54.4, respectively (including both observer and scientific cruise data).

**Table 1:** Description, mean, standard deviation (SD), and range of environmental parameters used in this study.

Parameter (Units)	Description	Mean	SD	Min	Max
DATATYPE	Dummy variable denoting data was from NOAA observers or tagging cruises (0 = observer, 1 = tagging)				
LON	Longitude of longline set	−90.38	2.85	−96.92	−81.27
LAT	Latitude of longline set	26.56	1.38	20.05	29.52
HKDPTH	Estimated depth of hooks	60.1	14.6	24	110
YEAR	Year of longline set	1999	3.9	1993	2005
MONTH	Month of longline set denoted by 3 dummy variables (MAR, APR, MAY). Month is June if all dummy variables are set to 0.				
SSHA	Sea surface height anomaly at longline set	−6.6	10.2	−33.5	37.6
SST	Sea surface temperature at longline set	25.4	4.1	20.5	29.9
BATHY	Bathymetry at longline set (Log-transformed)	7.52	0.27	3.61	8.27
BATHYSLP	Bathymetry slope at longline set (Log-transformed)	2.83	0.68	−0.37	5.31
EKE	Eddy kinetic energy at longline set (Log-transformed)	5.98	0.44	3.41	7.55
SSTSLP	SST slope at longline set (Log-transformed)	−4.09	0.67	−6.04	−1.55
WIND	Wind speed at longline set	6.07	1.54	2.71	10.74

All categorical variables (DATATYPE and MONTH) are denoted by dummy variables with values of 0 or 1.

All other variables are continuous variables and are normalized by subtracting the mean from the actual values prior to analysis.

Seven environmental variables were used as predictor variables in this study - sea surface temperature (SST), SST gradient (SSTSLP), sea surface height anomaly (SSHA), eddy kinetic energey (EKE), surface wind speed (WIND), bathymetry (BATHY), and bathymetric gradient (BATHYSLP). Details on the environmental data used in this study can be obtained from Teo et al. [20]. The environmental conditions associated with each longline set was determined as the 3×3 pixel mean around the starting location of each set.

Gridded SSTs from 1993 to 2005 in the GOM were extracted from the Pathfinder SST dataset (v5, http://www.podaac.jpl.nasa.gov). The data grids consisted of 8-day and monthly averaged SSTs on a 4-km equal angle grid. We preferred 8-day grids but the monthly grid was used if the cloud cover within 1° of the longline set location was >50%. In addition, SST gradients were calculated by performing a two-dimensional convolution on the GOM bathymetry grid with a 3×3 Sobel filter [23], [24].

We downloaded SSHA and geostrophic velocity anomaly data, which were derived from merged satellite altimetry measurements of four satellite altimetrs (Jason-1, ENVISAT/ERS, Geosat Follow-On and Topex/Poseidon interlaced) (AVISO, http://www.aviso.oceanobs.com). The SSHA and geostrophic velocity data extended from 1993 to 2005, with data assimilated every 7 days. Sea surface height anomalies are a direct way of identifying eddies, with negative SSHA indicating the presence of cyclonic eddies. Geostrophic velocity anomalies during the breeding season (March-June) were used to calculate the EKE in the GOM. Eddy kinetic energy is a commonly used measure of the mesoscale variability of the flow in a region and helps to identify regions where mesoscale eddies and current meanders are relatively common [25], [26], [27].

Ocean surface wind speed data were downloaded from the ERS-1/2 and QuikSCAT scatterometers (http://www.oceanwatch.pfeg.noaa.gov). From 1993 to 1999, we used wind speed data from the ERS-1/2 and from 2000–2005, we used data from the QuikSCAT satellite. All of the wind speed data consisted of 8-day averaged grids.

Bathymetry for the GOM was extracted from the Smith and Sandwell dataset v8.2 [28], which is a 2′ by 2′ global topographic dataset derived from ship soundings and satellite altimetry data (http://topex.ucsd.edu/marine_topo). A preliminary examination of the bathymetry data found two small spurious islands at approximately 25.53N, 90.42W, which were removed before analysis. Bathymetry values for the deleted area were subsequently filled by interpolation from surrounding pixels.

For ease of comparison and analysis, we log-transformed and normalized the fisheries and environmental data in order to transform the predictor variables onto relatively similar scales. First, we log-transformed SSTSLP, EKE, BATHY, and BATHYSLP. Second, we normalized all the non-categorical predictor variables in our model (LAT, LON, YEAR, HKDPTH, SST, SSTSLP, SSHA, EKE, WIND, BATHY, and BATHYSLP) by subtracting the mean from the values. However, we did not normalize the set month because this categorical variable entered the model as three dummy variables (March, April, and May) (Table 1). In addition, we also included second-order predictor variables to account for dome or bowl-shaped responses to changing environmental conditions [20], [29] (Table 1).

### Models

The number of each species caught on a longline are considered as count variables (i.e., non-negative integer-valued variables) and consequently, count data models like Poisson and negative binomial models are often used to analyze these data [30], [31], [32]. In a standard Poisson model, which is the simplest count data model, the variance is assumed to be equal to the mean but real data often violates this assumption by having variances greater than the mean (i.e. overdispersed). Negative binomial models, which allow for the variance to differ from the mean, are often used to model count data when the data is found to be overdispersed [17]. Exploration of the longline data indicated that bluefin and yellowfin tuna catch were overdispersed and were better described by negative binomial models. Therefore, we decided to use negative binomial models to compare the environmental influences on both bluefin and yellowfin tuna catch.

For a negative binomial model, the expected number of fish, E(*Y*) for a given longline set, *i*, is equal to the mean, µ*_i_*, and is given by,
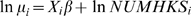
(1)where *X_i_* is a vector of explanatory variables (Table 1) for the *i*th set, *β* is a vector of parameters to be estimated, and *NUMHKS_i_* is the number of hooks used in set *i* (in thousands of hooks), which is an offset term to correct for the fishing effort of each set. The variance of the number of fish, var(*Y*), has an estimable shape parameter θ, and is given by,
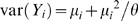
(2)therefore as θ^−1^ approaches zero, the variance approaches µ, and the negative binomial model becomes equivalent to a Poisson model. The probability of catching *Y* number of fish on set *i* is thus given by,
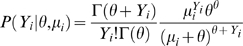
(3)which we can use to estimate the probability of catching more than one fish at a given location and ocean conditions.

For both bluefin and yellowfin models, we used a stepwise forward-backward model selection process with Akaike's Information Criterion with small sample correction (AICc) as the selection criterion [18]. This allowed us to select the best explanatory model by retaining explanatory variables that significantly improved the fit of the model while discarding those that do not. First, the numbers of bluefin or yellowfin tuna caught on each longline set were fit to a null model without any explanatory variables. Subsequently, at each step of the model selection process, each explanatory variable was in turn added to (if it was not yet in the model) or subtracted from (if it was already in the model) the model and fit to data. The variable that improved the AICc of the model most was retained in the model, and the process was reiterated until adding or subtracting variables did not improve the AICc, resulting in the final ‘best’ explanatory model [18]. In order to test the robustness of the final model, we also performed the model selection process from a full model with all the explanatory variables as well as five random starting models. In addition, we cross-validated the final models of both species using leave-one-out cross-validation and calculated the normalized root mean square deviations. Model fitting and selection was performed using the R language (v2.9.0) in conjunction with the MASS package [33].

The final selected models were used to determine the relative probability of catching bluefin and yellowfin tuna under environmental conditions of different years. This allowed us to visualize areas in the GOM where bluefin tuna were more likely to be caught on longlines. Environmental data grids were first downloaded for the GOM for each period (see above). The environmental data were then coupled with the bluefin and yellowfin models to determine the expected number of fish caught using equation 1 for each area in the GOM. Subsequently, equation 3 was used to calculate the relative probability of catching one or more bluefin or yellowfin tuna, given the environmental conditions in the area.

We also performed a sensitivity analysis on the final selected models to test the importance of each model parameter on the probability of catching bluefin and yellowfin in the GOM. First, we determined the probability of catching one or more bluefin and yellowfin in a single set of 1000 hooks, using the final bluefin and yellowfin models while keeping all parameters at their mean. Second, we perturbed each parameter in turn by one SD, while keeping the other parameters at their means, and calculated the probability of catching one or more fish. Third, we calculated the sensitivity of the model to each parameter as the percentage change in probability of catching one or more fish for each one SD of change in the parameter. Finally, we calculated the ratio of the proability of catching one or more yellowfin to bluefin. This ratio informs us of the parameter's influence on reducing bluefin bycatch without affecting yellowfin catch.

## Results

**Figure 3:**
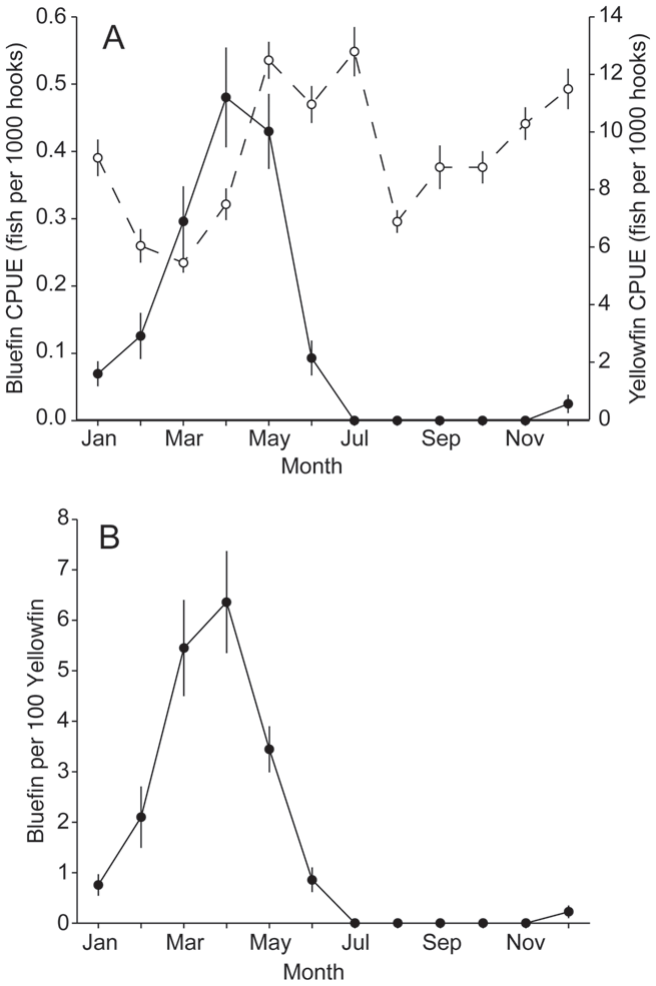
Mean and relative bluefin and yellowfin tuna CPUE. Circles indicate monthly mean (A) catch per unit effort (CPUE) of bluefin (closed circles) and yellowfin tuna (open circles) in the Gulf of Mexico, and (B) mean ratio of number of bluefin to 100 yellowfin caught. Error bars indicate 1 sd (based on 1000 bootstrap samples).

Bluefin tuna bycatch in the GOM was highly seasonal. The majority (87.4%) of observed bluefin bycatch occurred during the known bluefin breeding season from March to June (Fig. 3A). Peak bluefin CPUE occured in April (0.472±0.075 fish per 1000 hooks) and May (0.427±0.053 fish per 1000 hooks) while no bluefin tuna were caught in the GOM from July through November (Fig. 3A). In contrast, yellowfin CPUE was less variable, with yellowfin tuna being caught in the GOM throughout the year (Fig. 3A). Although yellowfin CPUE also showed seasonal variability with highest CPUEs occuring in July (12.8±0.84 fish per 1000 hooks) and lowest CPUEs in March (5.48±0.34 fish per 1000 hooks), the seasonal variability of yellowfin CPUE was substantially less than for bluefin tuna (Fig. 3A). The coefficient of variability (CV) of yellowfin CPUE by month (0.26) was also substantially lower than that for bluefin tuna (1.38). Even if we include only the bluefin breeding season from March to June, the CV of bluefin tuna CPUE by month (0.53) was still much higher than for yellowfin. Since bluefin CPUE was more variable than yellowfin CPUE, variability in the bluefin bycatch rate relative to yellowfin catch was primarily determined by variability in bluefin CPUE (Fig. 3B). Bluefin bycatch rate relative to yellowfin CPUE was highest in April (6.4±1.0 bluefin caught per 100 yellowfin) (Fig. 3B).

**Figure 4:**
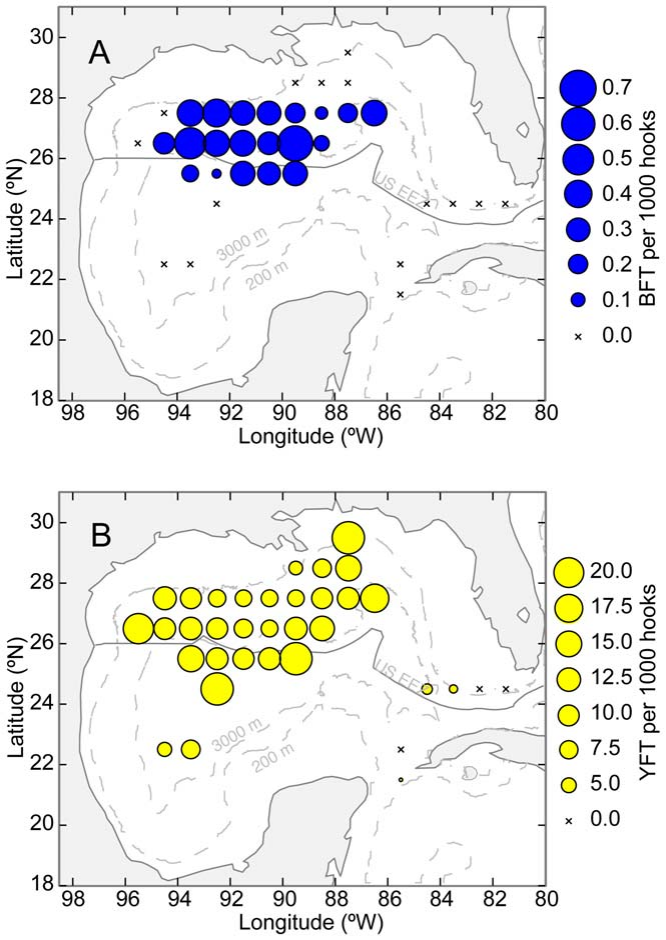
Spatial variability of bluefin and yellowfin tuna CPUE. Catch per unit effort (CPUE) of (A) bluefin and (B) yellowfin tuna are indicated by size of circles. Crosses indicate locations where more than 5000 hooks were set but no fish were caught.

The spatial range of bluefin tuna bycatch in the GOM appeared to be more limited than the spatial range of yellowfin tuna catch. Yellowfin tuna were caught in some areas (e.g., northeastern GOM) where bluefin tuna were not caught (Fig. 4, S1 & S2). In particular, the central and western GOM appeared to have relatively higher bluefin CPUE than other areas, especially in March and April (Fig. S1). In contrast, yellowfin tuna were caught in most areas observed throughout the year (Fig. S2). Importantly, bluefin CPUE was not significantly correlated yellowfin CPUE between March and June (r = −0.03, p = 0.35).

After the model selection process, the respective negative binomial models fit the bluefin and yellowfin tuna data well. The AICc for the bluefin and yellowfin null negative binomial models were 1392.3 and 5637.4 respectively. By including environmental and biological variables during the model selection process, the fit of the models were improved substantially (bluefin AICc: 1179.2, yellowfin AICc: 5262.7). In addition, we compared the negative binomial models with Poisson models and found that negative binomial models had better fits to the data than the respective Poisson models (bluefin AICc: 1206.6, yellowfin AICc: 6946.8). We performed a cross-validation of the final models and the normalized root mean square deviation was estimated to be 10.8 and 11.9% for the bluefin and yellowfin models respectively.

**Figure 5:**
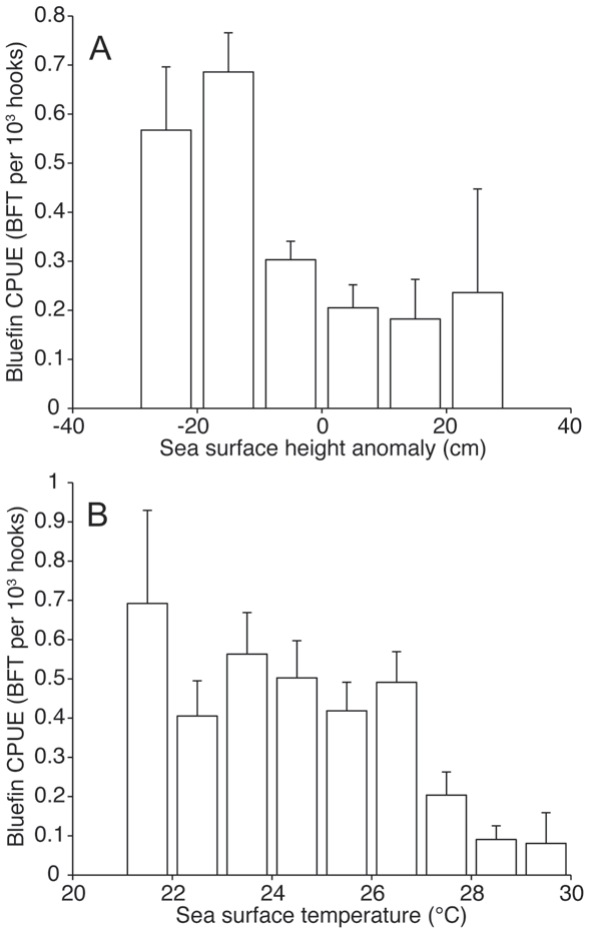
Bluefin tuna CPUE in relation to sea surface height anomaly and sea surface temperature. Histograms show mean catch per unit effort (CPUE) of bluefin tuna in the Gulf of Mexico, with respect to (A) sea surface height anomaly, and (B) sea surface temperature of the longline set. Error bars indicate 1 sd (based on 1000 bootstrap samples).

**Figure 6:**
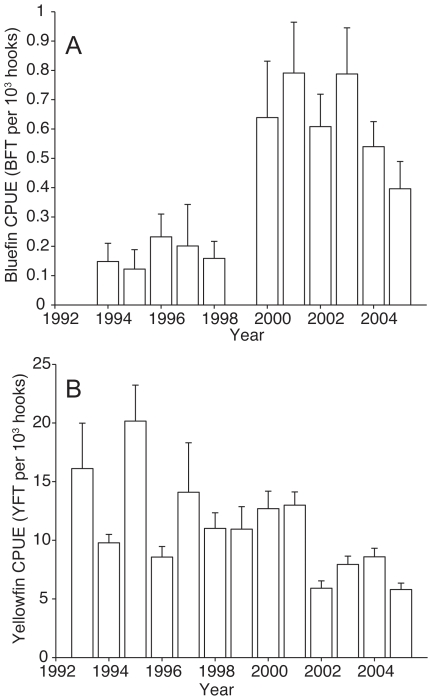
Annual bluefin and yellowfin tuna CPUE. Histograms show annual mean catch per unit effort (CPUE) of (A) bluefin and (B) yellowfin tuna in the Gulf of Mexico, from 1993 to 2005. Error bars indicate 1 sd (based on 1000 bootstrap samples).

The variables (and their estimated coefficients) in the final selected bluefin and yellowfin models after the model selection process can be seen in Tables 2 and 3, respectively. In the bluefin model, the SST, SSHA, EKE, and WIND were retained as important environmental variables (Table 2). Of particular interest, areas in the GOM with negative SSHAs and cooler SSTs were significantly correlated with higher bluefin CPUE (SSHA: r = −0.11, p<0.001; SST: r = −0.12, p<0.001) (Fig. 5). The negative coefficient associated with WIND2 (second-order wind speed) indicates a dome-shaped response of bluefin CPUE to wind speed (Table 2). The relationship between BATHY and bluefin CPUE also suggested that bluefin tuna are primarily caught off the continental shelf in relatively deep waters (Table 2). Latitude also strongly affected bluefin CPUE but longitude did not (Table 2). Bluefin catches were primarily restricted to an area between 25 to 28°N. Interestingly, there was a large increase in bluefin CPUE from 2000 to 2005 as compared to the preceding period (Fig. 6). The NOAA began prohibiting the use of live bait in August 2000, we therefore determined if the use of live bait affected bluefin and yellowfin CPUE. However, we found that the use of live bait did not significantly affect bluefin CPUE (z = −0.352, p = 0.725) but significantly increased yellowfin CPUE (z = 3.193, p = 0.001). In addition, due to the deliberate targeting of bluefin tuna for tagging, the scientific tagging cruises had a higher bluefin CPUE but lower yellowfin CPUE as compared to commercial longline sets (Table 2 & 3).

**Table 2:** Parameters and estimated coefficients of final bluefin tuna model.

	Estimated Coefficient	Standard Error	Z value	ΔAICc
DATATYPE	1.27	0.225	5.65	26.8
LAT	0.474	0.206	2.30	3.7
LAT2	−0.894	0.179	−4.99	58.0
YEAR	0.102	2.35E-2	4.33	18.2
YEAR2	−3.22E-2	6.88E-3	−4.69	19.8
MONTH (APR)	0.470	0.199	2.36	3.4
MONTH (MAY)	0.614	0.204	3.02	7.4
BATHY	0.930	0.395	2.353	4.2
SST	−0.177	5.52E-2	−3.21	8.6
SSHA	−2.42E-2	1.06E-2	−2.28	3.3
SSHA2	−9.20E-4	6.09E-4	−1.51	0.5
EKE	−0.661	0.203	−3.26	8.8
WIND2	−5.62E-2	3.48E-2	−1.61	0.8
INTERCEPT	−7.58	0.227	−33.4	
THETA	1.22	0.328		
2 log L	−1148.7			
AICc	1179.2			

ΔAICc is the difference in model AICc if parameter is excluded from model.

Z value is the ratio of estimated coefficient to standard error.

Parameters with a 2 are 2nd order environmental parameters.

**Table 3:** Parameters and estimated coefficients of final yellowfin tuna model.

	Estimated Coefficient	Standard Error	Z value	ΔAICc
DATATYPE	−0.622	0.140	−4.45	18.4
LON	−0.174	2.02E-2	−8.64	75.7
LON2	9.46E-3	4.12E-3	2.30	3.0
LAT	0.335	4.85E-2	6.92	46.4
LAT2	3.39E-2	1.53E-2	2.21	3.1
HKDEPTH	3.60E-3	2.25E-3	1.60	0.6
YEAR	−4.35E-2	7.99E-3	−5.44	27.2
YEAR2	−5.50E-3	2.25E-3	−2.55	3.9
MONTH (APR)	0.147	8.24E-2	1.79	1.2
MONTH (MAY)	0.348	6.52E-2	5.33	25.6
BATHY	0.688	9.88E-2	6.96	49.3
BATHYSLP2	6.71E-2	3.13E-2	2.15	2.8
EKE	0.255	7.87E-2	3.24	8.5
EKE2	0.141	5.21E-2	2.71	5.1
WIND	−0.179	2.10E-2	−8.55	67.6
INTERCEPT	−5.14	8.33E-2	−61.8	
THETA	1.879	0.124		
2 log L	−5227.968			
AICc	5262.591			

ΔAICc is the difference in model AICc if parameter is excluded from model.

Z value is the ratio of estimated coefficient to standard error.

Parameters with a 2 are 2nd order environmental parameters.

**Figure 7:**
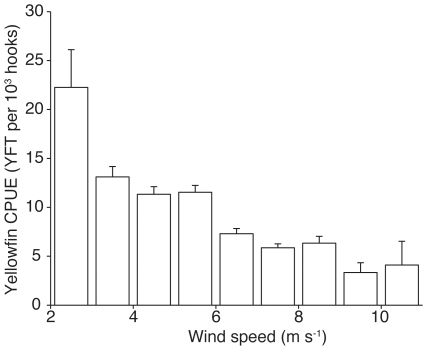
Yellowfin tuna CPUE in relation to wind speed. Histograms show mean catch per unit effort (CPUE) of yellowfin tuna in the Gulf of Mexico, with respect to wind speed of the longline set. Error bars indicate 1 sd (based on 1000 bootstrap samples).

In the yellowfin model, SST and SSHA were not retained as important environmental variables. Instead, EKE and WIND were retained as important environmental variables in the final selected model (Table 3). Areas with lower wind speeds were significantly correlated with higher yellowfin CPUE (r = −0.31, p<0.001) (Fig. 7). The positive coefficient associated with EKE2 (second-order EKE) indicates a bowl-shaped response to EKE (Table 3). The yellowfin CPUE was also affected by BATHY and BATHYSLP2 (second-order BATHYSLP), with higher yellowfin CPUE in deeper waters (Table 3). Unlike bluefin tuna, yellowfin tuna were affected by both longitude and latitude of the longline set (Table 3). Yellowfin CPUE have also declined slightly in the GOM in recent years (Table 3 & Fig. 6).

**Figure 8:**
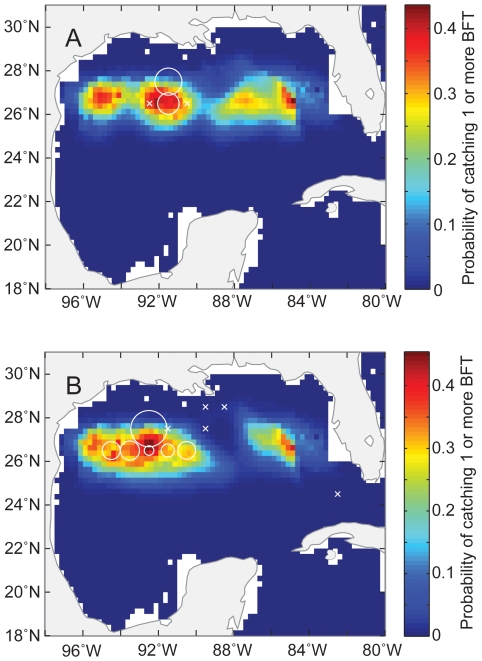
Expected probability of catching bluefin tuna. Colors indicate the expected probability of catching one or more bluefin tuna in the Gulf of Mexico on 15 May (A) 2002 and (B) 2005. Circles indicate actual relative bluefin tuna CPUE for May 2002 and 2005. Crosses indicate locations where at least one longline set was deployed but no fish were caught.

**Figure 9:**
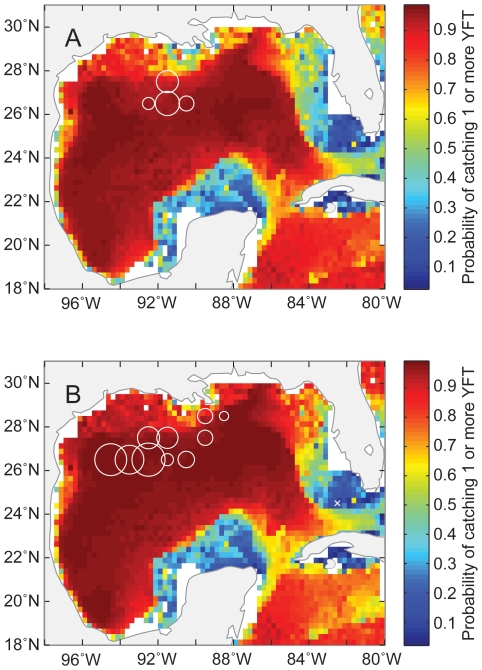
Expected probability of catching yellowfin tuna. Colors indicate the expected probability of catching one or more yellowfin tuna in the Gulf of Mexico on 15 May (A) 2002 and (B) 2005. Circles indicate actual relative yellowfin tuna CPUE for May 2002 and 2005. Crosses indicate locations where at least one longline set was deployed but no fish were caught.

**Figure 10:**
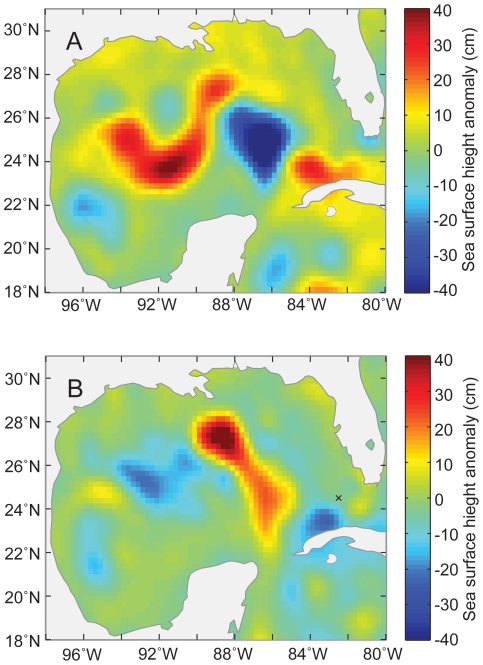
Map of sea surface height anomalies. Colors indicate sea surface height anomalies on 15 May (A) 2002 and (B) 2005.

We can use these two models to estimate the probability of catching bluefin and yellowfin tuna in the GOM. As an example, we have shown the estimated probability of catching bluefin and yellowfin tuna for May in 2002 and 2005 in Figures 8 and 9, and compared that with the SSHA (Fig. 10) and actual CPUE during those periods. Areas with a higher probability of catching bluefin tuna appeared to be associated with cyclonic eddies (Fig. 8). In contrast, it appears that the entire GOM basin have a relatively high probability of catching yellowfin tuna, other than the continental shelf, especially in the eastern GOM (Fig. 9).

Our sensitivity analysis indicated that the probability of catching bluefin tuna was most sensitive to the latitude of the longline set (Table 4). A one SD perturbation of the latitude southwards results in a 86.7% reduction of the proability of catching bluefin tuna. Importantly, SST and SSHA were the strongest dynamic environmental influences on bluefin bycatch rates, while WIND had only a relatively small influence. Bathymetry and EKE were also important environmental variables but these are temporally static variables that do not change through time. In contrast to bluefin tuna, the probability of catching yellowfin tuna in an area appeared to be relatively insensitive to changes in the model parameters. (Table 4). A one SD perturbation in any model parameter affected the probability of catching yellowfin tuna by less than 10%. In order to reduce the bycatch of bluefin tuna while minimizing impacts on yellowfin tuna catches, we looked at the ratio of the probability of catching yellowfin tuna relative to bluefin tuna. Under average conditions, the probability of catching yellowfin is 2.67 times higher than bluefin tuna. However, with a pertubation of one SD in SSHA or SST, we can increase that ratio to 3.41 and 3.45 respectively, resulting in fewer bluefin tuna being caught as bycatch (Table 4).

**Table 4:** Sensitivity analysis of environmental parameters on the probability of catching one or more BFT and YFT.

Parameters	Percent change in P(BFT >0) (%)		Percent change in P(YFT >0) (%)		Ratio of P(YFT >0) to P(BFT >0)	
	+1 SD	−1 SD	+1 SD	−1 SD	+1 SD	−1 SD
DATATYPE	93.3		−9.46		1.25	
LON	0	0	−5.98	4.46	2.51	2.79
LAT	−55.8	−86.7	4.15	−5.38	6.30	19.0
HOOKDEPTH	0	0	0.549	−0.584	2.69	2.66
YEAR	−6.25	−49.3	−3.16	0.878	2.76	5.32
MONTH (MAR)	0		0		2.67	
MONTH (APR)	33.9		1.45		2.03	
MONTH (MAY)	44.8		3.04		1.90	
SSHA	−21.6	10.5	0	0	3.41	2.42
SST	−22.5	25.4	0	0	3.45	2.13
BATHY	35.0	−29.7	3.11	−4.47	2.04	3.62
BATHYSLP	0	0	0.475	0.475	2.69	2.69
EKE	−27.1	31.5	2.17	−1.23	3.75	2.01
WIND	−8.77	−8.77	−3.48	2.52	2.82	3.01

Setting the model to average conditions has a P(YFT >0):P(BFT >0) ratio of 2.67.

## Discussion

Our results show that there are seasonal patterns and oceanographic features that influence the probability of bluefin bycatch in the GOM. Bluefin tuna appear seasonally in the GOM for breeding, which may be a balance between the environmental conditions required for spawning and the physiology of mature bluefin tuna (e.g., thermal tolerance, cardiac performance), and the growth and survival of larval bluefin tuna [9]. Bluefin CPUE had very high seasonal variability, with highest CPUEs in the months of April and May, coinciding with the peak of the spawning season [14]. In contrast, yellowfin CPUE had relatively small seasonal variability, with a substantially lower CV. Bluefin tuna also appeared to have a smaller spatial distribution in the GOM relative to yellowfin tuna, with bluefin tuna tending to be in the western and central GOM and between 25–28°N. However, the larger spatial distribution of the yellowfin tuna in the GOM may be due to its much larger population size and density, which can be seen in the much higher CPUE of yellowfin tuna.

In addition to lesser seasonal variability, yellowfin CPUE also appeared to be less sensitive to environmental conditions than bluefin CPUE. This may be due to the smaller size and physiology of yellowfin tuna, which has better cardiac performance at warmer temperatures than bluefin tuna [34]. This was consistent with our previous study on yellowfin tuna in the GOM using electronic tags [21]. Weng et al [21] showed that yellowfin tuna in the GOM were limited by cooler waters below the mixed layer but were not affected by warmer SSTs. For every environmental parameter that affected both yellowfin and bluefin CPUE (BATHY, EKE, and WIND), bluefin CPUE was several fold more sensitive to variability in those parameters than yellowfin CPUE. Based on our yellowfin model, the probability of catching yellowfin appear to be heavily influenced by whether longline sets are on or off shelf, with off-shelf sets being more likely to catch yellowfin tuna.

In contrast, we show that bluefin CPUE was significantly higher in areas with negative SSHAs and cooler SSTs, which are characteristic of mesoscale cyclonic eddies. Cyclonic eddies have positive vorticity and are associated with cooler SSTs, shallower thermoclines, and enhanced primary and secondary production [35], [36], [37], [38], [39]. These cooler regions may be important for adult bluefin tuna because warm SSTs, coupled with large body sizes and increased activity during courtship and spawning, may result in increased metabolic demand and cardiac stress. In addition, the increased production in these areas may improve the growth and survival of larval bluefin. One of the key oceanographic characteristics of the central and western GOM are the cyclonic and anti-cyclonic eddies generated by the Loop Current, which travel to the western GOM [19]. The Loop Current sheds large anti-cyclonic eddies in the eastern GOM, and these anti-cyclonic eddies in turn generate cyclonic eddies as they move from east to west [19].

Similar to this study, we previously showed that breeding bluefin tuna preferred areas with mesoscale eddies. However, we could not previously distinguish if breeding bluefin tuna preferred cyclonic or anti-cyclonic eddies due to the relatively coarse spatial resolution of light-based geolocation with respect to mesoscale features [20]. With the improved spatial resolution of the fisheries data, this study showed that bluefin CPUE in the GOM tended to increase in areas with cyclonic eddies (negative SSHAs and cooler SSTs).

It is important to note the large increases in bluefin CPUE from 2000 to 2005. One potential cause of this increase could be that our scientific tagging cruises, which were conducted from 1998 to 2002 and targeted bluefin tuna, biased the CPUE higher during those years. However, even excluding data from the tagging cruises, the CPUE pattern remained similar (Fig. S3). Changes in fishing gear and/or regulations may have also affected the CPUE of this fishery [40]. In August 2000, NOAA prohibited the use of live bait on pelagic longlines in the GOM [41]. However, the use of live bait did not significantly affect bluefin CPUE but significantly increased yellowfin CPUE. Another possibility could be an increase in spawning stock biomass for the Atlantic bluefin tuna in the western Atlantic. However, recent stock assessments suggest that the spawning stock biomass have remained at approximately similar levels during this period of high CPUE [16]. Although we cannot be certain of the causes of the increased bluefin CPUE from 2000 to 2005, it is possible that targeting of bluefin tuna in the GOM may have increased during this period. Our results from scientific tagging cruises targeting bluefin tuna suggest that longline sets and gear can be adjusted to target bluefin tuna. Early tagging cruises had relatively low bluefin CPUE but after we understood the preferred ocean conditions for bluefin tuna, our bluefin CPUE increased considerably[8].

These species specific differences in seasonal variability and sensitivity to environmental influences in the GOM suggest that bluefin tuna bycatch can be reduced substantially by managing the spatial and temporal distribution of the pelagic longline effort. Importantly, yellowfin CPUE was uncorrelated with bluefin CPUE, which suggests that reductions in bluefin bycatch can probably be achieved without substantially impacting yellowfin catches. One possible spatiotemporal management strategy would be to design a limited time-area closure during the bluefin tuna breeding season, especially April and May [6]. The areas closed could be dynamic in nature, with closures limited to areas with cyclonic eddies. Although time-area closures based on dynamic environmental conditions have been successfully employed in some fisheries, it is also relatively difficult to execute implement and enforce [6]. Potentially, pelagic longline vessels can be monitored with the Vessel Monitoring System (VMS) and vessels in high-risk areas can be redirected to areas with lower bycatch risk and higher probability of yellowfin catch. However, if a dynamic time-area closure is not feasible, we would suggest a limited fixed time-area closure of the central and western GOM within the US EEZ that has the highest probability of bluefin tuna bycatch during the peak spawning season.

It is also important to develop a comprehensive management strategy to reducing bluefin tuna bycatch in order to improve the rebuilding effort for the stock. In addition to spatiotemporal management of the fishery, gear changes would also likely help with reducing bluefin bycatch. Most importantly, incentives should be provided to the fishermen so as to align their objectives towards a reduction of bluefin bycatch. For example, a set number of bluefin tuna could be allowed to be caught and landed from the GOM. If the fishery exceeds the allowed number of bluefin tuna, the fishery could be closed until the end of the bluefin spawning season. In order to help the fishermen reduce the number of encounters with bluefin tuna, maps can be derived from remotely sensed data to show where bluefin bycatch is less likely to happen and yellowfin CPUE is likely to remain high. The management of bluefin tuna in the GOM should also be considered in coordination with other fisheries targeting western Atlantic bluefin. Recent work on bluefin tuna otoliths [42], intra-muscle pollutants [43] and genetics [44], [45] have shown that most, if not all, of the bluefin tuna on the GOM spawning ground are of western origin. In contrast, bluefin tuna in the northwest Atlantic are often of mixed stock origin with fish from both western and eastern Atlantic spawning grounds [42]. In addition, Armsworth et al. [46] showed that an economically optimal strategy for managing bluefin tuna in the western Atlantic would be to reduce the catches in both the northwestern Atlantic and the GOM.

The strong effect of latitude on our bluefin model is likely due to the US pelagic longline fleet predominantly staying within the US EEZ in the GOM and the preference of bluefin tuna for deeper waters away from the continental shelf. Our analysis in this study is limited to the US pelagic longline fishery. However, we believe that Non-US longline fleets operating in Mexican and international waters in the GOM are known to catch bluefin tuna but we were not able to obtain data from those fisheries. In order to get a more complete understanding of bluefin bycatch in the GOM, future studies should attempt to obtain fisheries data from these non-US longline fleets. Another limitation of this study's results is that if environmental conditions in the GOM change so drastically such that future conditions range beyond the limits of the conditions in our dataset, our models would not be able to estimate the probability of catching bluefin or yellowfin tuna in those areas and periods.

One of the possible ways to improve our models is to include zero-inflation into our parametric models [30]. During our exploration of the dataset, we explored the use of zero-inflated models in modelling bluefin and yellowfin CPUE. We found that yellowfin model fits improved with zero-inflated negative binomial models. However, we found that bluefin model fit was not improved with a zero-inflated Poisson nor a zero-inflated negative binomial model. Therefore, in the interest of making the bluefin and yellowfin models comparable, we decided to use negative binomial models without zero-inflation for both yellowfin and bluefin tuna. Another possible modelling framework is to use generalized additive models (GAMs) rather than the parametric generalized linear models used in this study [47]. However, we found that while GAMs tended to have improved prediction skill, the model results were more difficult to understand and interpret for comparative purposes. Since comparing the bluefin and yellowfin models was the key to this study, we decided to use parametric models, which provided good fits to the data. Although we optimized the fit of our models, there remains substantial unexplained variability in both bluefin and yellowfin models. This is not surprising because we modeled the data on a set by set basis, which tends to increase the variability. In future studies, one way to reduce the variability of the data is to model the data on a trip by trip basis but our current dataset did not have enough data to allow us to do so.

In 2007 and 2008, the NMFS deployed observers onto the pelagic longline fleet in the GOM at a higher rate (70–80%) than usual (<8%), in order to provide improved estimates of bluefin bycatch [10]. That dataset would have been ideal for improving and validating our models in this study. Unfortunately, we were unable to obtain those data for this study. Hopefully, when these new data becomes available in the future, we would be able to improve and validate our models in a future study.

In this study, we determined and compared the environmental influences on bluefin and yellowfin CPUE in the GOM. The results of this study can be used to determine the probability of bluefin bycatch in the US EEZ in the northern GOM in relation to yellowfin CPUE. By incorporating the results of this study into their managment plans, the managers of bluefin and yellowfin tuna can help reduce bluefin bycatch and improve the CPUE of yellowfin tuna. This would help improve the rebuilding effort for the western Atlantic stock of bluefin tuna and ensure the long term viability of fishing for pelagic fish in the GOM.

## Supporting Information

Figure S1 Spatiotemporal variability of bluefin tuna CPUE. Crosses indicate locations where more than 1000 hooks were set but no bluefin tuna were caught.
(2.20 MB PDF)

Figure S2 Spatiotemporal variability of yellowfin tuna CPUE. Crosses indicate locations where more than 1000 hooks were set but no yellowfin tuna were caught.
(2.24 MB PDF)

Figure S3 Annual bluefin CPUE. Histograms show annual mean catch per unit effort (CPUE) of bluefin tuna in the Gulf of Mexico, from 1993 to 2005 (fishery observer data only). Error bars indicate 1 sd (based on 1000 bootstrap samples).
(0.23 MB PDF)
