# Supplementary material for: Comparative Influence of Ocean Conditions on Yellowfin and Atlantic Bluefin Tuna Catch from Longlines in the Gulf of Mexico
Source: PLoS One. 2010 May 28;5(5):e10756. doi: 10.1371/journal.pone.0010756 (PMC2878315; doi:10.1371/journal.pone.0010756)
Supplement: Figure S1 — Spatiotemporal variability of bluefin tuna CPUE. Crosses indicate locations where more than 1000 hooks were set but no bluefin tuna were caught. (2.20 MB PDF) [file pone.0010756.s001.pdf]

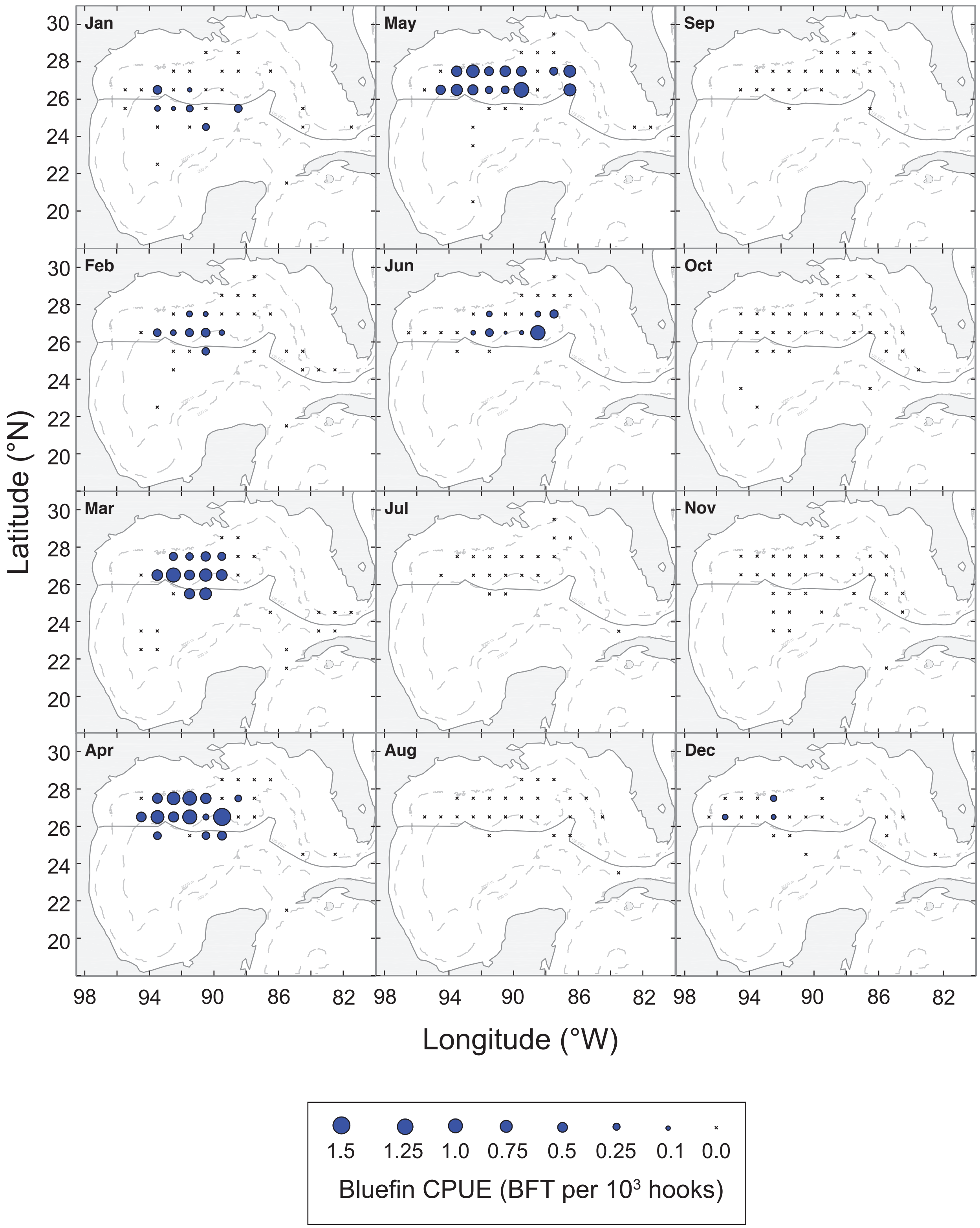

Figure S1. Spatiotemporal variability of bluefin tuna CPUE. Crosses indicate locations where more than 1000 hooks were set but no bluefin were caught.
