# Supplementary material for: Comparative Influence of Ocean Conditions on Yellowfin and Atlantic Bluefin Tuna Catch from Longlines in the Gulf of Mexico
Source: PLoS One. 2010 May 28;5(5):e10756. doi: 10.1371/journal.pone.0010756 (PMC2878315; doi:10.1371/journal.pone.0010756)
Supplement: Figure S3 — Annual bluefin CPUE. Histograms show annual mean catch per unit effort (CPUE) of bluefin tuna in the Gulf of Mexico, from 1993 to 2005 (fishery observer data only). Error bars indicate 1 sd (based on 1000 bootstrap samples). (0.23 MB PDF) [file pone.0010756.s003.pdf]

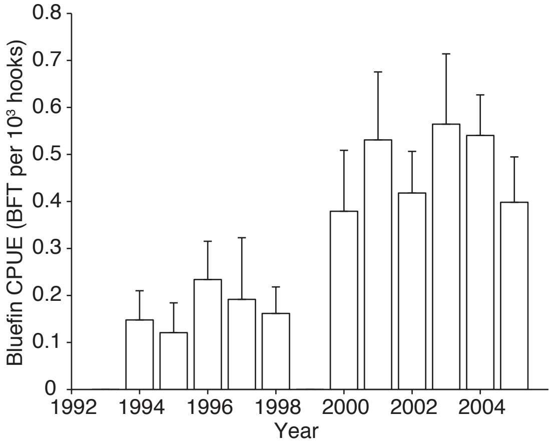

Figure S3. Annual bluefin CPUE. Histograms show annual mean catch per unit effort (CPUE) of bluefin tuna in the Gulf of Mexico, from 1993 to 2005 (fishery observer data only). Error bars indicate 1 sd (based on 1000 bootstrap samples).
